# Double dissociation of the effects of volitional control on perceptual selection and maintenance in multistable visual perception

**DOI:** 10.1167/jov.26.3.12

**Published:** 2026-03-23

**Authors:** David W. Bressler, Sean Noah, Andrew Lu, Michael A. Silver

**Affiliations:** 1 Herbert Wertheim School of Optometry & Vision Science, University of California, Berkeley, Berkeley, CA USA; 2 Department of Neuroscience, University of California, Berkeley, Berkeley, CA USA; 3 Helen Wills Neuroscience Institute, University of California, Berkeley, Berkeley, CA USA; 4 UC Berkeley Center for the Science of Psychedelics, University of California, Berkeley, Berkeley, CA USA; 5 Department of Molecular and Cell Biology, University of California, Berkeley, Berkeley, CA USA

**Keywords:** binocular rivalry, perceptual selection, multistable perception, Gestalt grouping

## Abstract

Stimuli that give rise to multistable percepts are powerful tools for understanding the psychological and neural processes and mechanisms that create conscious perception. Research on multistable perception has resulted in substantial insights regarding the stimulus and configural factors that influence perceptual interpretations of ambiguous stimuli and the modulation of these interpretations by contextual and cognitive factors. However, relatively little is known about the effects of subjects’ attempts to volitionally enhance conscious perception of one of the perceptual interpretations of a multistable stimulus. We used dichoptic presentation of Díaz-Caneja stimuli that allow four distinct perceptual interpretations: two monocular percepts (corresponding to the two monocular stimuli), and two interocularly-grouped percepts (each of which combine information from the two eyes). In different trials, participants were instructed to attempt to enhance predominance of one of the four perceptual interpretations. In contrast to the very limited effects of volitional control on conventional binocular rivalry between orthogonal gratings, we observed strong volitional enhancement of the predominance of the cued percept for Díaz-Caneja stimuli. This enhancement resulted mainly from longer mean dominance durations for the two monocular percepts and from increased probability of selection for the two interocularly-grouped percepts. A control experiment indicated that the effects of volitional control on perceptual predominance in binocular rivalry between Díaz-Caneja stimuli were unlikely to be due to response bias. These findings demonstrate distinct and dissociable processes underlying the effects of volitional control on perceptual selection and maintenance for multistable stimuli.

## Introduction

The study of visual awareness and its substrates in the brain has been greatly facilitated by the use of stimuli that are consistent with multiple coherent and enduring perceptual interpretations. These multistable stimuli often involve periods of dominance of one of the perceptual interpretations and simultaneous suppression of the other perceptual interpretations (Blake and Logothetis, 2002; Kim and Blake, 2005). Even though the multistable stimuli are static, there are frequently transitions from one perceptual interpretation of the ambiguous stimuli to another, with some aspects of the stimuli dominating perception at any given time, while others are rendered invisible.

Binocular rivalry is a particularly well-studied type of multistable perception (Tong, Meng, & Blake, 2006; Brascamp and Baker, 2013; Blake 2022). A typical binocular rivalry display involves presentation of two stimuli to corresponding locations in the two eyes, with each eye viewing a stimulus that cannot be perceptually fused with the other eye's stimulus. In general, the resulting perceptual experience is not a combination or a summation of the two incompatible stimuli or sustained dominance of one stimulus by the other. Rather, observers experience alternations between periods of perceptual dominance of one stimulus at a time, with the other stimulus being phenomenally suppressed.

These perceptual alternations in binocular rivalry are generally considered to be spontaneous and automatic, and they persist for the duration of presentation of the rivalrous pair of stimuli. However, perceptual dominance and suppression and their dynamics in binocular rivalry can be substantially influenced by factors such as stimulus configuration, spatial context, prior expectations, attention, and Gestalt grouping cues (reviewed in Bressler, Denison, & Silver, 2013).

One of these modulatory factors in conscious perception of ambiguous stimuli is volitional control, or the ability of the observer to willfully increase the dominance of one of the possible perceptual interpretations. Although there is evidence that observers can substantially change the rate of perceptual alternations in binocular rivalry, especially after practice, current evidence indicates relatively modest ability for subjects to volitionally enhance their perception of a given stimulus in binocular rivalry (Meng & Tong, 2004; van Ee, van Dam, & Brouwer, 2005).

In this study, we measured the ability of participants to volitionally increase dominance of one of four perceptual interpretations that are afforded by Díaz-Caneja stimuli. These stimuli, named after their discoverer, are rivaling horseshoe-shaped stimuli that point in opposite directions in the two eyes (Díaz-Caneja, 1928; translated into English in Alais, O'Shea, Mesana-Alais, & Wilson, 2000). When viewing these stimuli, one of the two monocular horseshoes is sometimes perceived, while at other times, the visual system groups information from the two monocular stimuli to create one of two additional possible perceptual interpretations: concentric circles or horizontal lines (Figure 3). Díaz-Caneja stimuli are therefore useful for understanding interocular competition and how it interacts with grouping processes to generate coherent visual percepts from ambiguous stimuli.

We found that compared to conventional binocular rivalry for pairs of gratings with orthogonal orientations, participants exhibited significant volitional control over their perceptual experiences of Díaz-Caneja stimuli. These effects of volitional control occurred through distinct mechanisms: perceptual enhancement of the monocular stimuli resulted primarily from prolonging the mean duration of periods of dominance of these stimuli after they had been perceptually selected, whereas enhancement of the interocularly-grouped percepts was mainly due to increased probability of perceptual selection. This double dissociation of perceptual selection and maintenance for two different types of perceptual interpretations of the same stimuli provides a foundation for further investigation of stimulus and cognitive factors that influence conscious experience and their associated neural correlates.

## Methods

### Subjects

The same group of nine subjects participated in all three experiments, and two additional subjects participated in both Experiments 1 and 2. A twelfth subject participated only in Experiment 2 but was not available for Experiment 1. All subjects had normal or corrected-to-normal vision. Three of the authors were subjects in all three experiments, and the other nine subjects were naive about the experiment(s). All subjects had previous experience as participants in binocular rivalry psychophysical experiments.

### Stimuli

Matlab and Psychophysics Toolbox (Brainard, 1997; Pelli, 1997; Kleiner et al., 2007) were used to generate the stimuli. Stimuli were presented on a gamma-corrected, NEC CRT FE992 monitor with screen resolution of 1280 × 1024 pixels and frame refresh rate of 60 Hz. The mean luminance of the stimuli and the background luminance were 59 cd/m^2^. Fixation points were in the form of plus signs that were located in the center of each of the stimuli.

Subjects viewed the stimuli through a stereoscope at a viewing distance of 100 cm, so that the stimulus to the left of the fixation point was presented only to the left eye, and the stimulus to the right of the fixation point was presented only to the right eye. The stereoscope was adjusted for each subject before the experiment to make sure that the two images were overlapping. Subjects used a head and chin rest to stabilize their head and reduce movement.

### Experiment 1

Subjects viewed a pair of dichoptically-presented gratings, one leftward-tilted (45° counterclockwise from vertical) and one rightward-tilted (45 degrees clockwise from vertical). Subjects were instructed to continuously maintain their gaze on the fixation point in the center of the stimuli. For each 60-second trial, subjects continuously viewed the stimuli and indicated their perception by pressing and holding one of two buttons corresponding to the leftward-tilted and rightward-tilted gratings. They were instructed to withhold their response if their percept was ambiguous.

There were three experimental conditions: passive viewing (12 trials), volitional enhancement of the leftward-tilted grating (24 trials), and volitional enhancement of the rightward-tilted grating (24 trials). The passive viewing condition was always presented first, and the order of the two subsequent blocks of volitional enhancement trials were counterbalanced across subjects. For volitional enhancement, subjects were instructed to attempt to increase the total dominance time of the cued grating orientation.

Spatial frequency of the gratings and temporal frequency of flicker of the gratings were also varied, but the results of these manipulations will be reported in another article. For the data presented here, the spatial frequency of the gratings were 7 cyc/°, and there was no flicker. The gratings had a Michelson contrast of 0.2 and a diameter of 1.8° of visual angle and were presented on a gray background. The orientations of the gratings presented to each eye were counterbalanced within each subject.

### Experiment 2

Subjects viewed a pair of dichoptically-presented horseshoe-shaped stimuli composed of gratings with a spatial frequency of 7 cyc/°, Michelson contrast of 0.2, and diameter of 1.8° of visual angle (Figure 3). The horseshoe stimuli were formed by bisecting and recombining two sine-wave luminance-modulated gratings, one of concentric circles and one of horizontal lines, along the vertical line bisecting the stimuli.

Subjects were instructed to fixate on the fixation point in the center of the stimuli and to indicate their percept by continuously pressing and holding one of four buttons on a keypad. The buttons corresponded to the four possible unitary percepts (leftward-pointing horseshoe, rightward-pointing horseshoe, concentric circles, and horizontal lines; Figure 3). In the case of ambiguous perception, subjects were instructed to withhold a response. On a given trial, subjects either passively viewed the stimuli or were instructed to attempt to increase the total dominance time of one of the four unitary percepts. The volitional instructions were presented on the screen at the beginning of each trial.

Each participant completed eight passive-viewing trials and two each of volitional selection of each of the four perceptual interpretations. In other trials, subjects were instructed to attempt to increase the total dominance duration of either (leftward- and rightward-horseshoe percepts) or (concentric circles and horizontal lines percepts). Results from those trials will be reported in a separate article. The orientations of the horseshoe stimuli presented to each eye were counterbalanced within each subject.

### Experiment 3

To determine whether any observed volitional enhancement of predominance could be explained by a tendency to press the key for the cued perceptual interpretation irrespective of perceptual content (response bias), we conducted a control experiment in which the monocular inputs were either (i) dichoptic monocular horseshoes (stimulus set 1; same stimuli as Experiment 2) or (ii) concentric circles in one eye and horizontal lines in the other (stimulus set 2). In the latter case, grouping cues that lead to perception of concentric circles and horizontal lines are present in each the monocular stimuli, resulting in a low predominance of perception of interocularly-grouped horseshoe percepts. Therefore any measured “volitional enhancement” of horseshoe dominance for stimulus set 2 would likely be due to response bias.

The stimuli used in Experiment 3 were otherwise identical to those in Experiment 2 (same spatial frequency, contrast, and stimulus size). Each participant completed ten trials for stimulus set 1 and ten for stimulus set 2. For each of the stimulus sets, there were two passive-viewing trials, four of volitional selection of leftward-pointing horseshoes, and four of volitional selection of rightward-pointing horseshoes. The duration of each trial was 60 seconds.

## Results

In Experiment 1, participants attempted to enhance perception of one of two gratings of orthogonal orientations presented as a rivalrous pair. We then tested the effects of volitional control over one of four possible perceptual interpretations of Díaz-Caneja stimuli (Experiment 2; Figure 3). Finally, we conducted a control experiment (Experiment 3) to estimate possible effects of response bias on the measures of the effects of volitional control in Experiment 2.

### Experiment 1: Effects of volitional control on predominance in binocular rivalry between orthogonal gratings

Previous work reported limited ability of observers to exert volitional control over their conscious perception in conventional binocular rivalry between two incompatible stimuli (Meng & Tong, 2004; van Ee et al., 2005). In Experiment 1, we measured volitional control for binocular rivalry between two orthogonal gratings. Subjects continuously viewed dichoptically-presented grating pairs for 1-minute trials through a mirror stereoscope, and they reported their percepts in real time.

Specifically, subjects pressed one of two buttons at the beginning of each period of unitary perception of either the leftward- or rightward-tilted grating, and they held down the button for as long as that percept was maintained. They were instructed to withhold any response for ambiguous percepts that were not exclusively one grating orientation or the other (piecemeal mixture of the two orientations, overlap or summation of the two stimuli resulting in a plaid, etc.). For each one-minute trial, the total dominance times of perception for leftward- and rightward-tilted gratings were calculated.

**Figure 1.**
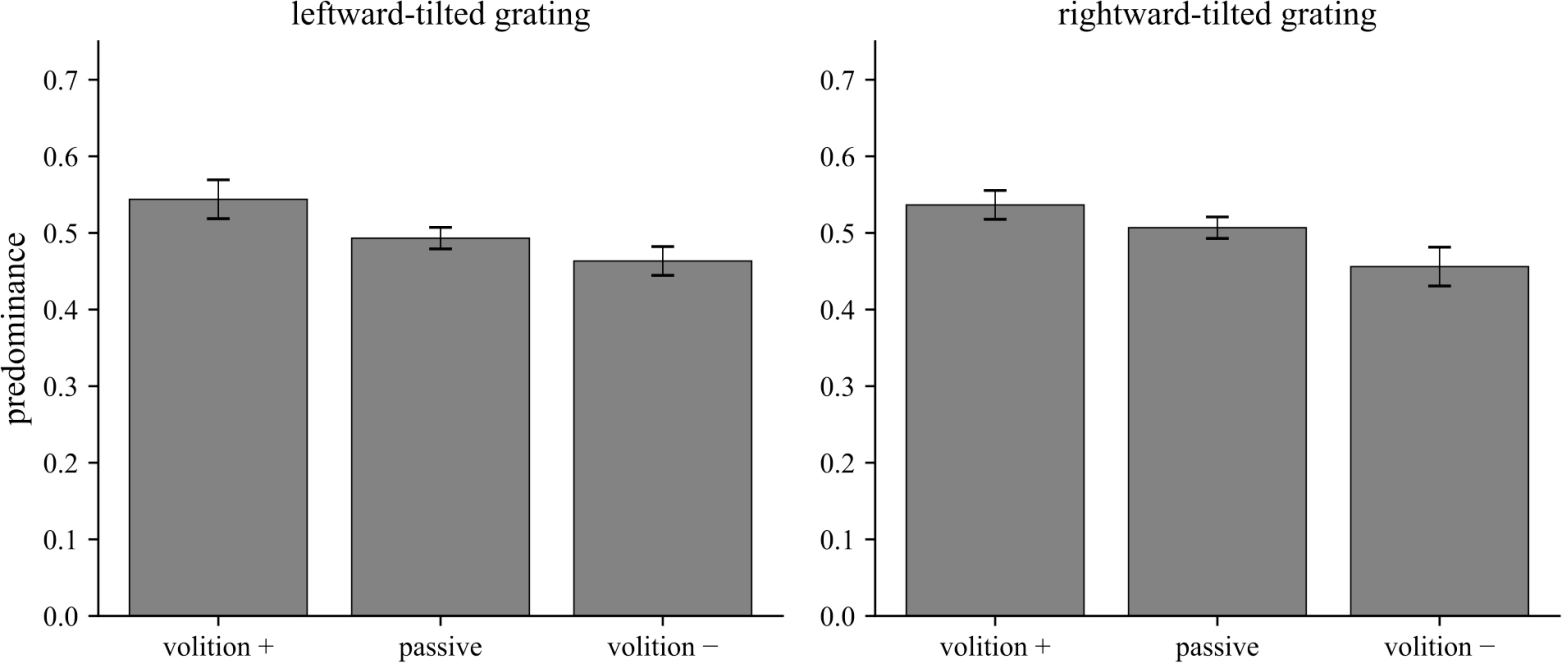
Volitional control of predominance in conventional binocular rivalry. Subjects were instructed to attempt to increase the total dominance time of one of two gratings of orthogonal orientations that were dichoptically presented in a binocular rivalry display. volition +: the orientation that subjects were attempting to enhance; volition −: the orthogonal orientation to volition +; passive: no attempt to exert volitional control over which orientation was perceived. Relative to passive viewing, volitional control did not significantly affect perceptual predominance.

Before each trial, subjects were instructed to enhance their perception of the leftward-tilted grating, to enhance their perception of the rightward-tilted grating, or to passively view the stimuli. Predominance of perception is shown in Figure 1 for each orientation—when it was volitionally selected (volition +), when it was orthogonal to the orientation that was volitionally selected (volition −), and during passive viewing (passive). For each of these three conditions, predominance of perception was defined as the total dominance time for perception of a given orientation divided by the total dominance time for any unitary perception (either leftward- or rightward-tilted grating).

For conventional binocular rivalry between orthogonal gratings, there are only two possible unitary percepts. We found that attempting to volitionally enhance perception of one of the orientations resulted in a small and nonsignificant increase in predominance for the cued orientation, relative to passive viewing. For leftward-tilted gratings, mean predominance for volition + was 0.545, compared to 0.493 during passive viewing [Δ = 0.051; 95% confidence interval (CI) = −0.012 to 0.114; *t*(10) = 1.82; one-tailed *t*-test *p* = 0.049; *p* = 0.099 following Holm-Bonferroni correction across the two *t*-tests, one for each orientation; Cohen's *d* = 0.55].

For rightward-tilted gratings, mean predominance for volition + was 0.537, compared to 0.507 during passive viewing [Δ = 0.030; 95% CI = −0.021 to 0.082; *t*(10) = 1.32; one-tailed *t*-test *p* = 0.108; *p* = 0.108 following Holm-Bonferroni correction; Cohen's *d* = 0.40]. Overall, these results indicate very limited volitional control over predominance for binocular rivalry between orthogonal gratings. These findings are generally consistent with those of Meng and Tong (2004) and van Ee et al. (2005) for binocular rivalry between orthogonal gratings and between face and house stimuli.

### Experiment 1: Effects of volitional control on mean dominance duration and probability of selection in binocular rivalry between orthogonal gratings

**Figure 2.**
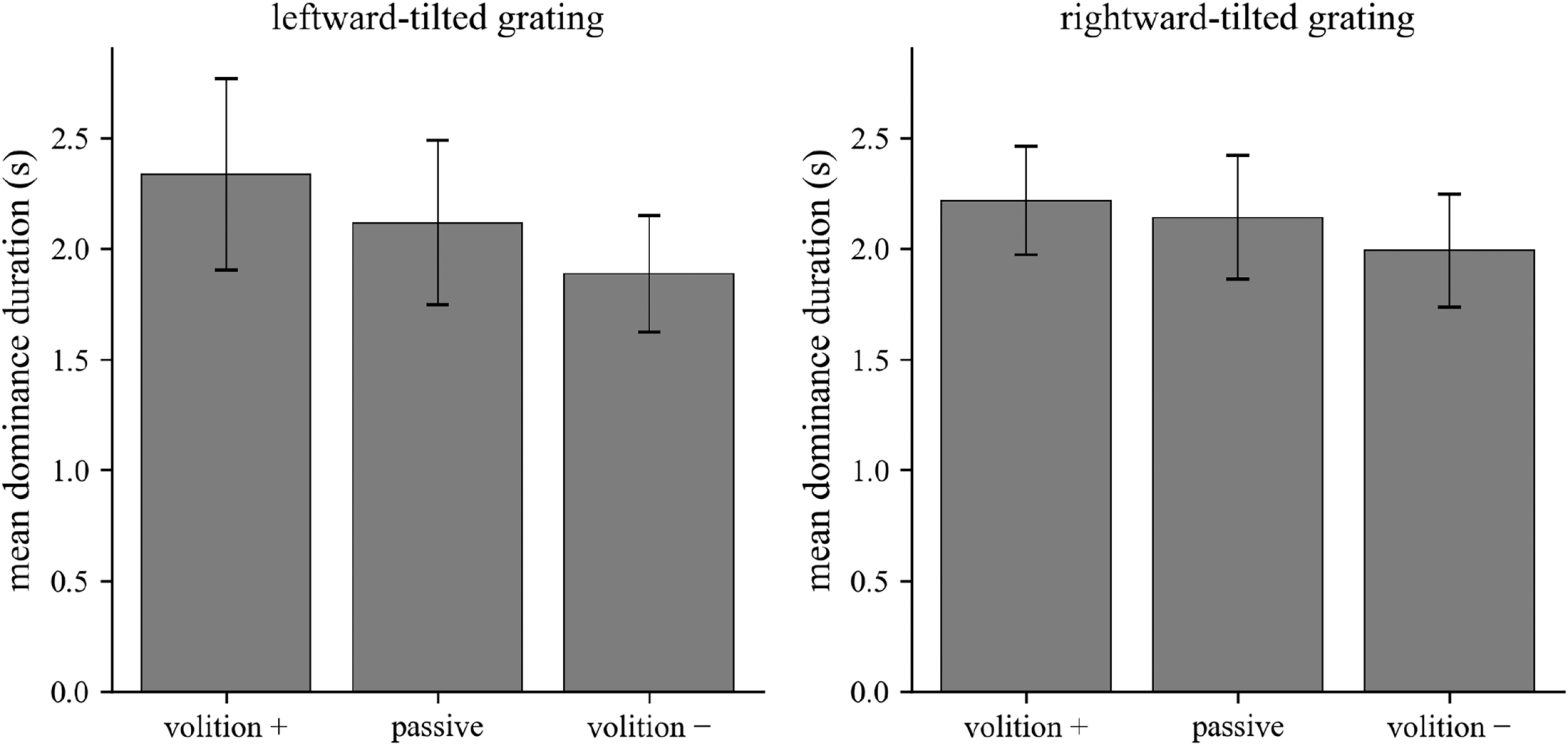
Volitional control did not significantly affect mean dominance duration (volition +) in conventional binocular rivalry between orthogonal gratings, relative to passive viewing. Experimental conditions are the same as those in Figure 1.

Figure 2 displays mean dominance durations for each orientation when it was volitionally selected (volition +), when it was orthogonal to the one that was volitionally selected (volition −), and during passive viewing (passive).

For the leftward-tilted grating, mean dominance duration for volition + (2.334 s) was not significantly different from passive viewing (2.116 s) [Δ = 0.217 s; 95% CI = −0.496 to 0.931 s; *t*(10) = 0.68; *p*(one-tailed) = 0.256, Holm-Bonferroni-corrected *p* = 0.513; Cohen's *d* = 0.20]. For the rightward-tilted grating, the corresponding comparison was also not significant [volition + = 2.216 s; passive = 2.140 s; Δ = 0.076 s; 95% CI = −0.277 to 0.429 s; *t*(10) = 0.48; *p*(one-tailed) = 0.320; Holm-Bonferroni-corrected *p* = 0.513; Cohen's *d* = 0.15].

We next examined the probability of selection for each grating orientation. Probability of selection was defined as the number of dominance periods for a given orientation divided by the total number of unitary dominance periods (perception of either a leftward- or rightward-tilted grating). We then tested, for each orientation, whether the probability of selecting the cued orientation (volition +) exceeded a value of 0.5.

For the leftward-tilted grating, probability of selection in the volition + condition was 0.508 (95% CI = 0.492–0.524), which did not significantly exceed a value of 0.5 [*t*(10) = 1.14; *p*(one-tailed) = 0.141; Holm-Bonferroni-corrected *p* = 0.282; Cohen's *d* = 0.34]. For the rightward-tilted grating, the probability of selection in the volition + condition was 0.488 [95% CI = 0.477 to 0.500; t(10) = −2.23; *p*(one-tailed) = 0.975; Holm-Bonferroni-corrected *p* = 0.975; Cohen's *d* = −0.67].

### Experiment 2: Relative predominance of the four possible percepts in binocular rivalry between Díaz-Caneja stimuli

**Figure 3.**
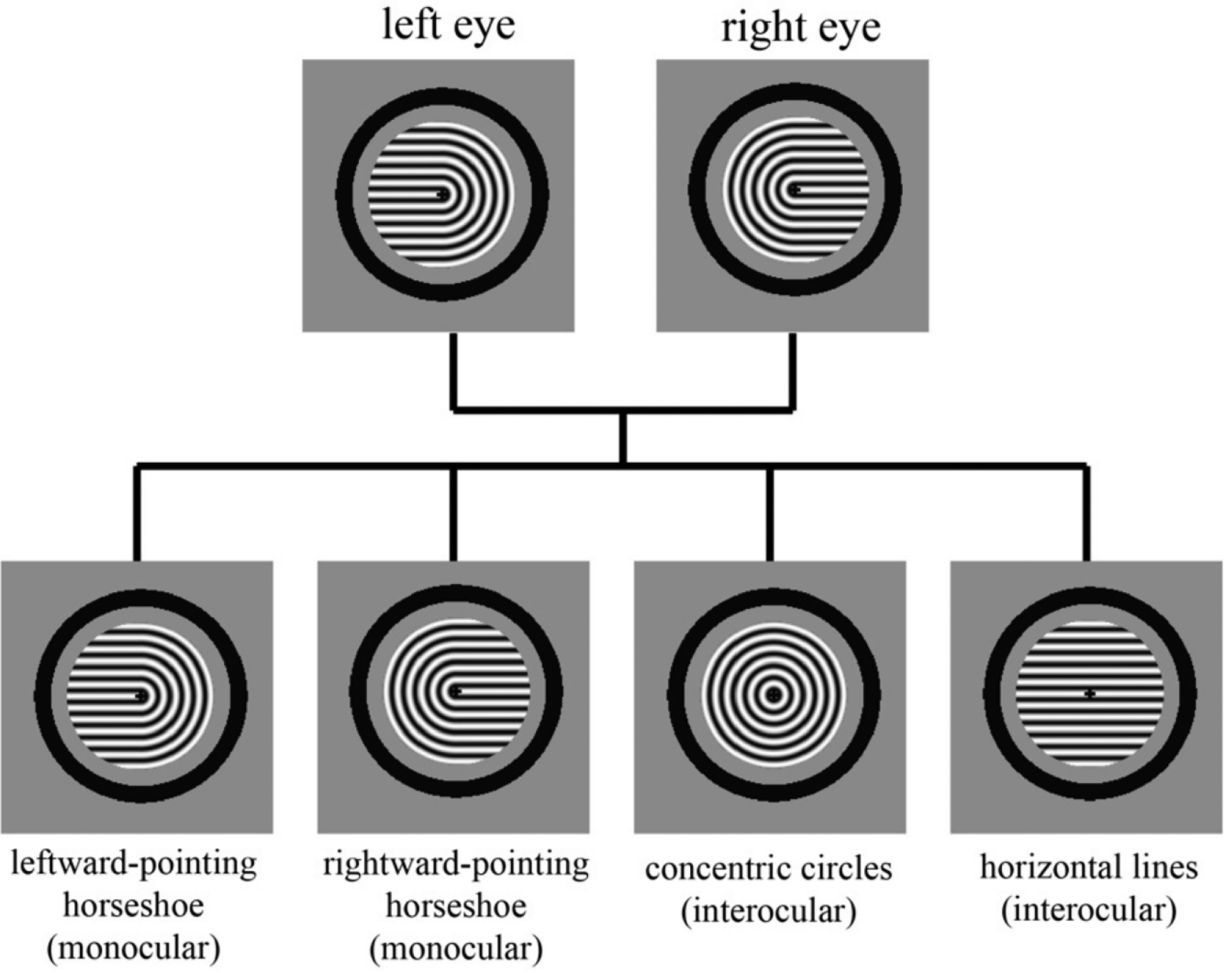
Díaz-Caneja stimuli and the four possible associated unitary percepts. Top row: Díaz-Caneja stimuli. The rivalrous pair consists of two horseshoes that point in opposite directions in the two eyes. These can also be presented in the opposite configuration, with the rightward-pointing horseshoe in the left eye and the leftward-pointing horseshoe in the right eye. Bottom row: four possible unitary percepts arising from viewing of Díaz-Caneja stimuli.

The results of Experiment 1 indicate very limited volitional control over which of two competing percepts dominates in conventional binocular rivalry between gratings with orthogonal orientations. In Experiment 1, volitional effects were not statistically significant for any of the three experimental measures: overall predominance, mean dominance duration, and probability of perceptual selection. In Experiment 2, we used a similar experimental design to characterize volitional effects on perception of Díaz-Caneja stimuli (Figure 3).

Díaz-Caneja stimuli are consistent with four distinct perceptual interpretations. Two of these (leftward-pointing and rightward-pointing horseshoes) correspond to the monocular stimuli and presumably result mainly from dominance of one eye's input and simultaneous suppression of the other eye's input. Interocular grouping of portions of each of the monocular inputs can result in two additional percepts: concentric circles and horizontal lines. These interocularly-grouped percepts likely result from interactions between stimulus representations that occur at a higher level in the visual system than the sites of competition between monocular inputs (Knapen, Paffen, Kanai, & van Ee, 2007).

As in Experiment 1, participants passively viewed rivalrous stimuli or were instructed to attempt to enhance one of the unitary perceptual interpretations. On different one-minute trials, these volitional efforts were directed towards enhancement of perception of the leftward-pointing horseshoe, the rightward-pointing horseshoe, concentric circles, or horizontal lines.

**Figure 4.**
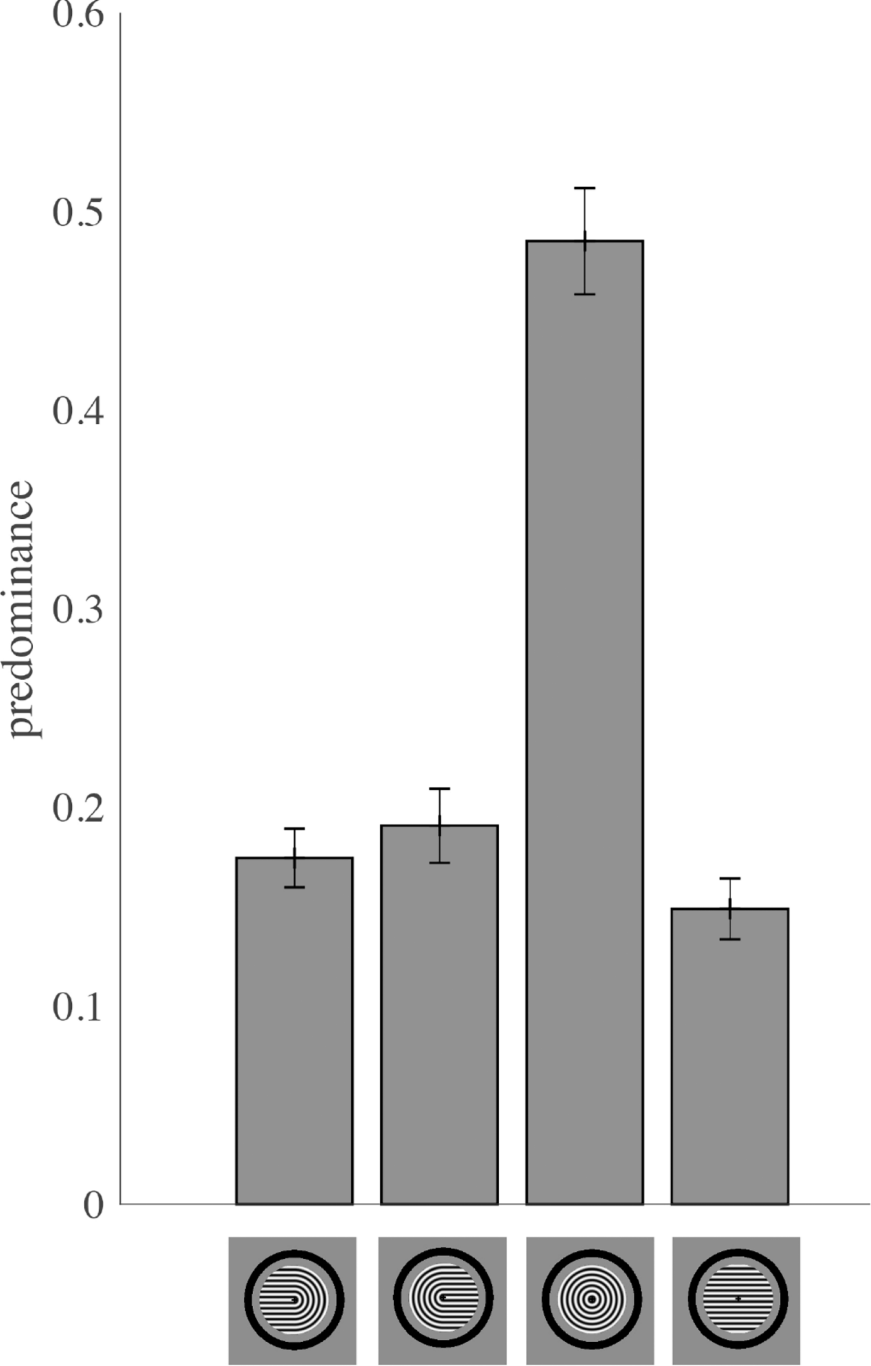
Relative predominance of the four unitary percepts during passive viewing of Díaz-Caneja stimuli. Perception of concentric circles was much more likely than that of the other three percepts.

Passive viewing of Díaz-Caneja stimuli resulted in significant amounts of predominance of each of the four unitary perceptual interpretations, with substantially more predominance for concentric circles (Figure 4). As in Experiment 1, predominance of a given percept was defined as the total dominance time for that percept divided by the total dominance time for all possible unitary percepts.

If all four perceptual interpretations had equal amounts of total dominance time, the predominance value for each one would be 0.25. For passive viewing, concentric circles showed the highest mean predominance (0.487), a value that was much greater than that of each of the other percepts: leftward-pointing horseshoe (0.173), rightward-pointing horseshoe (0.190), and horizontal lines (0.150).

Two-tailed two-sample *t*-tests (with Holm-Bonferroni correction for the three comparisons) confirmed that predominance of the concentric circles percept was greater than predominance of each of the other percepts (Holm-Bonferroni-corrected *p* values between 7.96 × 10⁻^5^ and 6.11 × 10⁻^6^), with large effect sizes (Cohen's *d* = 2.05–2.80). All other pairwise comparisons were not significant (Holm-Bonferroni-corrected *p* values > 0.52).

A previous study of perception of Díaz-Caneja stimui reported approximately equal predominance of monocular (combining the two horseshoe percepts) and interocularly-grouped (combining concentric circles and horizontal lines) percepts (Ngo, Miller, Liu, & Pettigrew, 2000). However, this study did not report predominance values for the four individual percepts.

### Experiment 2: Mean dominance durations and probabilities of selection of the four possible percepts in binocular rivalry between Díaz-Caneja stimuli

**Figure 5.**
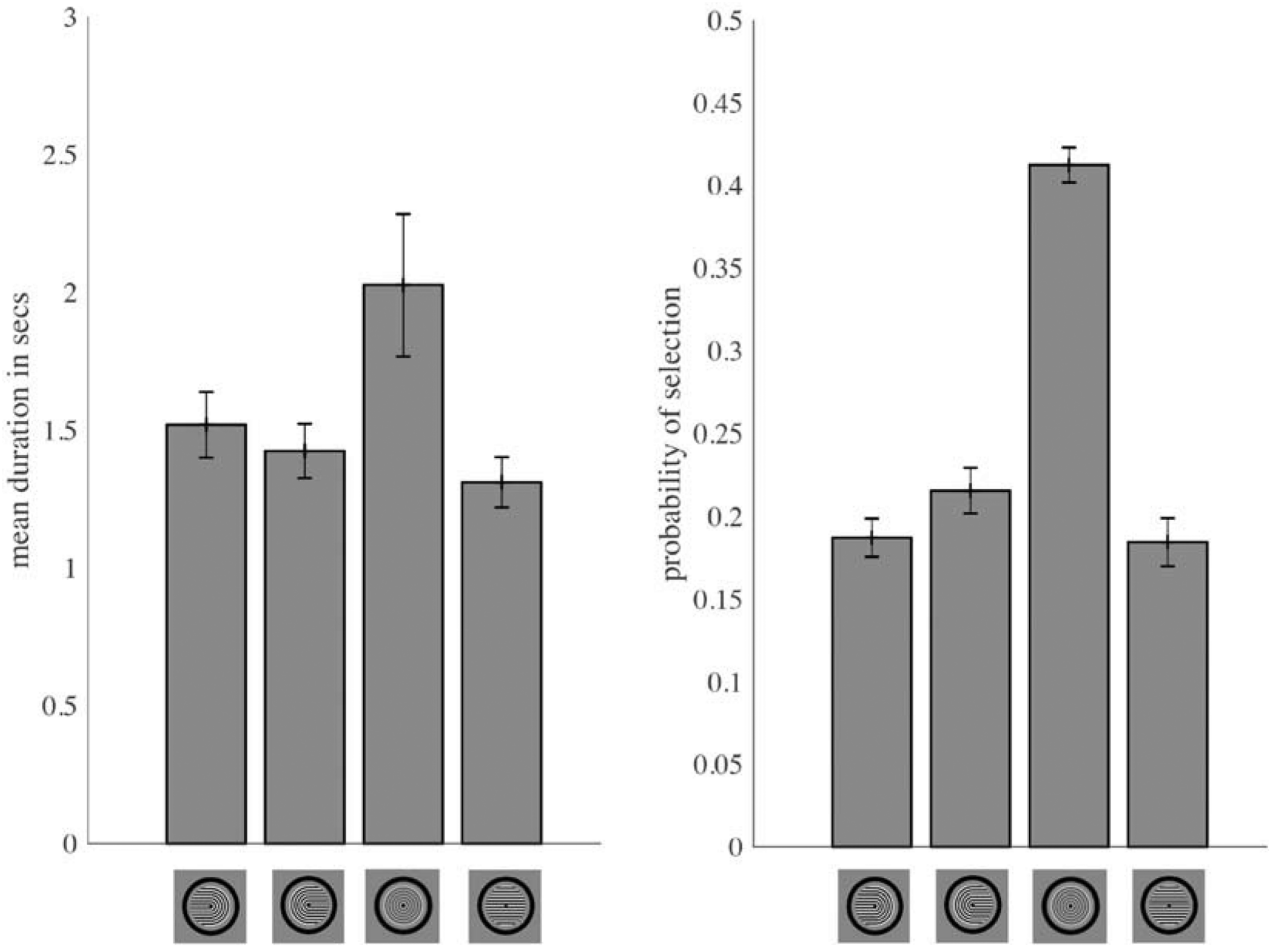
Mean dominance duration and probability of selection of the four possible percepts for passive viewing of Díaz-Caneja stimuli. The overall predominance of concentric circles is due primarily to increased probability of selection, relative to the other three percepts.

Predominance of a given percept is a function of both the mean dominance duration for that percept and its probability of selection. For passive viewing of Díaz-Caneja stimuli, we observed no significant difference in mean dominance duration between circles and the other three percepts (all Holm-Bonferroni-corrected *p* values ≥ 0.08; Figure 5). In contrast, the probability of perceiving concentric circles was significantly higher than that of each of the other percepts (all Holm-Bonferroni-corrected *p* values < 2.08 × 10⁻^6^; Cohen's *d* values = 2.9–3.5), whereas all other pairwise comparisons of probability of selection were not significant (all Holm-Bonferroni-corrected *p* values > 0.46) (Figure 5). Thus the higher predominance of perception of concentric circles in passive viewing of Díaz-Caneja stimuli is driven by a higher probability of selection of the concentric circles percept.

### Experiment 2: Effects of volitional control on predominance in binocular rivalry of Díaz-Caneja stimuli

**Figure 6.**
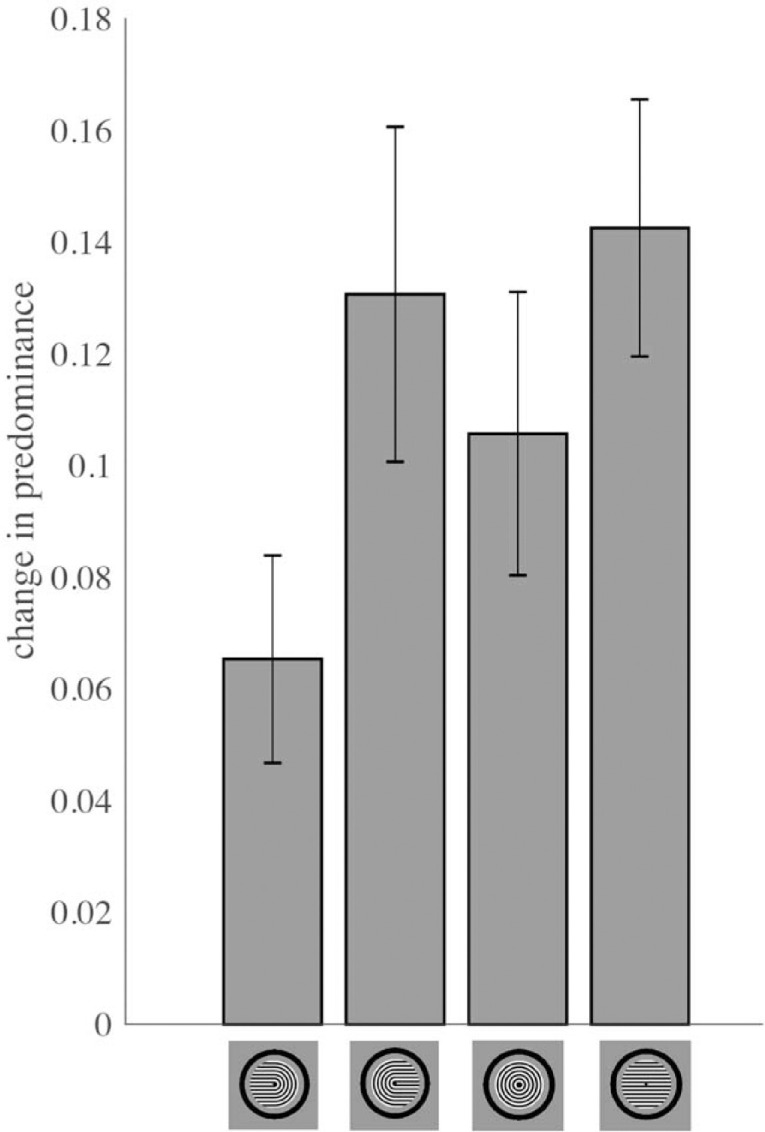
Volitional control increases predominance of the cued percept compared to passive viewing for all four perceptual interpretations of Díaz-Caneja stimuli.

In contrast to the very limited effects of volitional control for rivalry of pairs of orthogonal gratings (Experiment 1; Meng & Tong, 2004), we observed substantial effects of volitional control on predominance of perception for Díaz-Caneja stimuli (Figure 6). Relative to passive viewing, volition increased predominance for each of the four perceptual interpretations [concentric circles: Δ = 0.107; 95% CI = 0.051–0.162; *t*(11) = 4.24; one-tailed *t*-test *p* = 6.9 × 10⁻⁴; Holm-Bonferroni-corrected *p* = 2.07 × 10^−^^3^, Cohen's *d* = 1.23; horizontal lines: Δ = 0.142; 95% CI = 0.094–0.19; *t*(11) = 6.46; *p* = 2.3 × 10⁻⁵; Holm-Bonferroni-corrected *p* = 9.3 × 10⁻⁵; Cohen's *d* =1.87; leftward-pointing horseshoe: Δ = 0.076, 95% CI = 0.032 to 0.12; *t*(11) = 3.82; *p* = 1.43 × 10^−3^; Holm-Bonferroni-corrected *p* = 2.07 × 10^−3^; Cohen's *d* =1.10; rightward-pointing horseshoe: Δ = 0.127, 95% CI = 0.059 to 0.195; *t*(11) = 4.10; *p* = 8.8 × 10⁻⁴; Holm-Bonferroni-corrected *p* = 2.07 × 10^−3^; Cohen's *d* = 1.18].

Across the four types of perception of Díaz-Caneja stimuli, the maximum corrected *p*-value for volitional enhancement of predominance was 2.07 × 10^−3^, and the minimum Cohen's *d* was 1.10, indicating large effect sizes. Thus volitional enhancement of predominance was substantial for all four perceptual interpretations of Díaz-Caneja stimuli.

To quantify differences between Experiments 1 and 2 in the effects of volitional control on perceptual predominance, we measured the magnitude of volitional enhancement of predominance across the two experiments within participants (the difference between volition + and passive viewing). Because there are only two perceptual interpretations in Experiment 1 and four in Experiment 2, we normalized volitional effects on predominance using the following formula:

$$\begin{array}{ccc} \;\Delta \mathrm{Pred}\_\mathrm{norm}\; & = & \left(\mathrm{Pred}\_\mathrm{volitional}-\mathrm{Pred}\_\mathrm{passive}\right)/\\ & & \left(1-\mathrm{Pred}\_\mathrm{passive}\right) \end{array}$$

This normalized delta expresses volitional enhancement of perception as the proportion of the maximum possible increase for that perceptual interpretation (maximum predominance = 1.0). We then compared Experiment 2 to Experiment 1 within participants using paired *t*-tests on these normalized delta values. To equate the number of possible percepts in the two experiments in our analysis, we grouped leftward- and rightward-tilted gratings for Experiment 1 and (concentric circles and horizontal lines) or (leftward- and rightward-pointing horseshoes) for Experiment 2.

For concentric circles and horizontal lines, the mean difference between Experiments 1 and 2 across subjects in normalized volitional effects on predominance was 0.115 [95% CI = 0.052–0.178; *t*(10) = 4.06; two-tailed *t*-test *p* = 2.29 × 10^−3^; Holm-Bonferroni-corrected *p* = 6.86 × 10^−3^]. For horseshoe percepts, the mean difference between the two experiments was 0.055 [95% CI = −0.045 to 0.156; *t*(10) = 1.23; two-tailed *t*-test *p* = 0.249; Holm-Bonferroni-corrected p = 0.249].

### Experiment 2: Effects of volitional control on mean dominance duration and probability of selection in binocular rivalry of Díaz-Caneja stimuli

**Figure 7.**
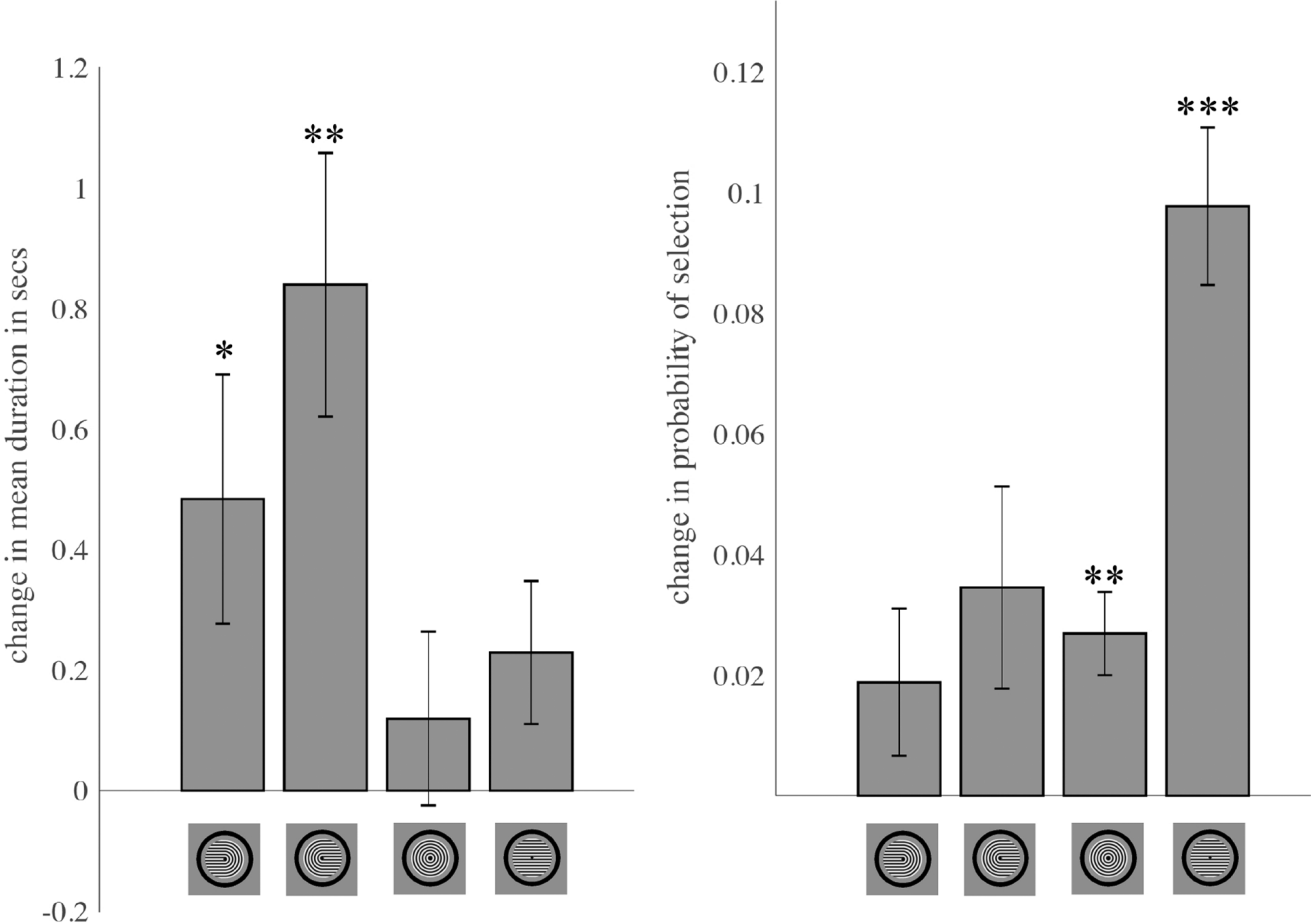
Volitional control primarily results in increased mean dominance duration of the selected percept for horseshoe (monocular) percepts and increased probability of selection for concentric circles and horizontal lines (interocularly-grouped) percepts.

Increases in predominance of a given perceptual interpretation due to volition could be due to an increase in mean dominance duration and/or an increase in the probability of selection. We observed that volitional enhancement of predominance of the monocular horseshoe percepts was mainly driven by an increase in mean dominance duration (i.e., prolonged maintenance of the volitionally selected percept) [Figure 7; leftward-pointing horseshoe: Δ = 0.549 s; 95% CI = 0.073–1.025 s; t(11) = 2.54; *p* = 0.0276; Holm-Bonferroni-corrected *p* = 0.0830; Cohen's *d* = 0.73; *p* = 0.039; rightward-pointing horseshoe: Δ = 0.892 s; 95% CI = 0.344–1.439 s; *t*(11) = 3.59; *p* = 0.0043; Holm-Bonferroni-corrected *p* = 0.017; Cohen's *d* = 1.04].

In contrast, volition did not have significant effects on mean dominance duration for interocularly-grouped percepts [concentric circles: Δ = 0.136 s; 95% CI = −0.160 to 0.432 s; *t*(11) = 1.01; *p* = 0.334; Holm-Bonferroni-corrected *p* = 0.334; Cohen's *d* = 0.29; horizontal lines: Δ = 0.219 s; 95% CI = 0.015–0.452 s; *t*(11) = 2.06; p = 0.0637; Holm-Bonferroni-corrected *p* = 0.127; Cohen's *d* = 0.60].

We observed the opposite pattern of results for probability of selection: volitional enhancement of interocularly-grouped concentric circles and horizontal lines occurred mainly through increases in probability of selection of the cued perceptual interpretation [concentric circles: Δ = 0.0243; 95% CI = 0.0071–0.0416; *t*(11) = 3.10; *p* = 0.0101; Holm-Bonferroni-corrected *p* = 0.0302; Cohen's *d* = 0.90; horizontal lines: Δ = 0.0974; 95% CI = 0.0727–0.122; *t*(11) = 8.70; *p* = 2.93 × 10⁻⁶; Holm-Bonferroni-corrected *p* = 1.17 × 10⁻⁵; Cohen's *d* = 2.51].

However, volition did not significantly affect probability of selection of monocular horseshoe perceptual interpretations [leftward-pointing horseshoe: Δ = 0.0259; 95% CI = −0.0077 to 0.059; t(11) = 1.70; *p* = 0.118; Holm-Bonferroni-corrected *p* = 0.141; Cohen's *d* = 0.49; rightward-pointing horseshoe: Δ = 0.0342; 95% CI = −0.0033 to 0.0717; t(11) = 2.01; *p* = 0.0702; Holm-Bonferroni-corrected *p* = 0.141; Cohen's *d* = 0.58].

To statistically test for a double dissociation between the type of volitional effect (mean dominance duration versus probability of selection) and percept type (monocular versus interocularly-grouped), we conducted a 2 × 2 repeated-measures analysis of variance with factors of Volitional Effect (the difference between volition and passive viewing for mean dominance duration versus probability of selection) and Percept Type (monocular horseshoes versus interocularly-grouped concentric circles/horizontal lines).

For each subject, delta values were *z*-standardized within each Volitional Effect to place outcomes on a common scale. The repeated-measures analysis of variance revealed a significant Volitional Effect × Percept Type interaction [*F*(1, 11) = 12.28, *p* = 0.0049, partial η² = 0.528, with no significant main effect of either Volitional Effect (*F* ≈ 0, *p* = 1.00) or Percept Type (*F* = 0.20, *p* = 0.663)]. This significant interaction indicates that volitional control acts at different stages of processing for the two percept types.

We further characterized this interaction with two-tailed *t*-tests on the *z*-standardized delta values. For monocular percepts, the increase caused by volition was greater for mean dominance duration than for probability of selection (*t*(11) = 2.34, Holm-Bonferroni-corrected *p* = 0.039, Cohen's *d* = 0.67), whereas for interocularly-grouped percepts, volition increased the probability of selection more than it increased mean dominance duration (*t*(11) = 3.55, Holm-Bonferroni-corrected *p* = 0.009, Cohen's *d* = 1.03). The double dissociation of Volitional Effect and Percept Type provides evidence that volitional enhancement primarily prolongs mean dominance duration for monocular percepts while primarily increasing probability of selection for interocularly-grouped percepts.

### Experiment 3: Control experiment to assess response bias

To assess whether the volitional enhancement of percepts in Experiment 2 could be explained by response bias, we conducted a control experiment with two stimulus sets. In stimulus set 1, the monocular stimuli were horseshoes as in Experiment 2 (i.e., Díaz-Caneja stimuli). For passive viewing of these stimuli, the predominance of horseshoe percepts was 0.338, and the predominance of concentric circles/horizontal lines percepts was 0.662.

**Figure 8.**
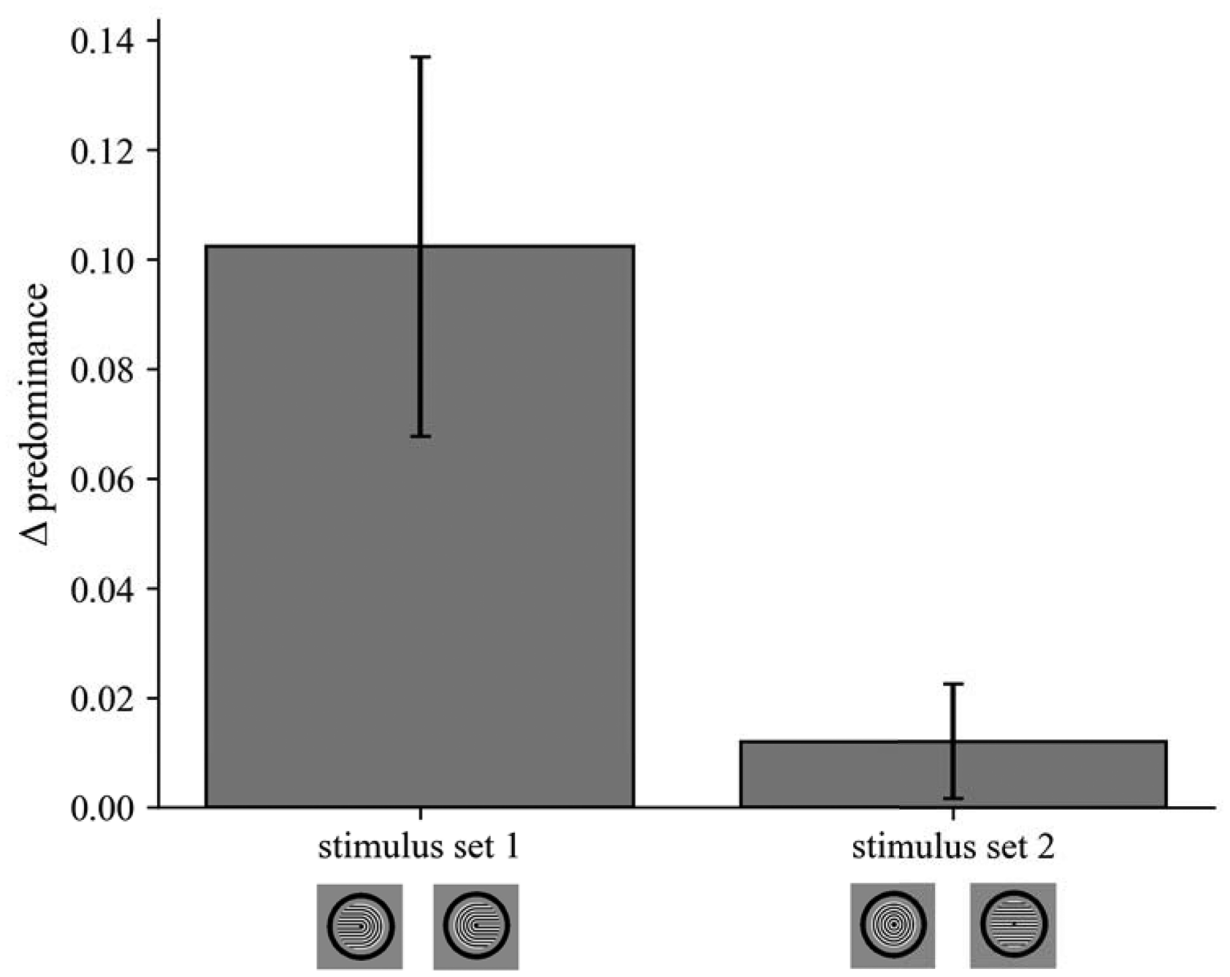
Results of Experiment 3 (control experiment to assess response bias). Change in predominance of perception of horseshoes (ΔPredominance; difference between volition + and passive viewing) was measured for two stimulus sets: set 1, in which the monocular stimuli were horseshoes, and set 2, in which the monocular stimuli were concentric circles and horizontal lines. For each stimulus set, positive values indicate increased predominance of horseshoe percepts when they were volitionally cued, compared to passive viewing.

In stimulus set 2, the monocular stimuli were concentric circles and horizontal lines that cannot be easily perceptually combined into interocularly-grouped horseshoes, as both monocular stimulus differences and grouping cues encourage perception of concentric circles or horizontal lines (Figure 8). Indeed, for passive viewing of stimulus set 2, the predominance of horseshoe percepts was 0.170, and the predominance of circles/lines percepts was 0.830.

For both stimulus sets, participants passively viewed the stimuli or attempted to volitionally enhance predominance of either the leftward- or rightward-pointing horseshoe percept. Importantly, the instructions to the participants and the four response categories were identical for the two stimulus sets, so any effects of response bias should be similar for both stimulus sets. Similarly, any observed differences in volitional effects between the two stimulus sets indicate differences in conscious perception rather than in response bias.

For each participant and stimulus set in Experiment 3, we computed predominance as the fraction of time spent in horseshoe percepts divided by the total time of unitary perception and defined ΔPredominance as volition + minus passive viewing. The measure of ΔPredominance was significantly greater than zero for stimulus set 1 [mean = 0.102, 95% CI = 0.023–0.182; *t*(8) = 2.96; *p* = 0.018; Holm-Bonferroni-corrected *p* = 0.036; Figure 8]. These results indicate reliable volitional enhancement for Díaz-Caneja stimuli and are consistent with the findings from Experiment 2 for these stimuli. In contrast, ΔPredominance did not differ from zero for stimulus set 2 [mean = 0.012, 95% CI = −0.012 to 0.036; t(8) = 1.15; *p* = 0.284; Holm-Bonferroni-corrected *p* = 0.284; Figure 8].

A paired *t*-test across participants for the two experiments confirmed that the volitional effect on predominance was larger in stimulus set 1 than stimulus set 2 [set 2 − set 1: mean difference = −0.090, 95% CI = −0.164 to 0.016; *t*(8) = −2.82; *p* = 0.023]. Together, these results argue against a simple response bias account of the effects of volitional control on predominance in binocular rivalry between Díaz-Caneja stimuli that were found in Experiment 2.

## Discussion

We used multistable perception to study the contributions of interocular interactions and stimulus grouping to conscious perception of dichoptic Díaz-Caneja stimuli. We also instructed participants to attempt to enhance their total dominance time of one of the four perceptual interpretations in order to characterize the effects of volitional control. Multistable perception, and binocular rivalry in particular, offers a powerful means of better understanding the psychophysical and neural processes underlying generation of conscious perception from ambiguous sensory inputs (Blake & Logothetis 2002; Tong et al., 2006; Blake, 2022) and allows differentiation of those processes associated with perceptual selection versus those underlying maintenance of a given percept once it has attained dominance (Levelt, 1965; Alais & Blake, 1999; Ooi & He, 2003; Silver & Logothetis, 2004; de Weert, Snoeren, & Koning, 2005; Alais, Lorenceau, Arrighi, & Cass, 2006; Bressler, et al., 2013).

### Multiple levels of perceptual selection in binocular rivalry

Although binocular rivalry is often characterized as arising from interocular conflict between stimuli presented to the two eyes that cannot be fused into a coherent and stable percept, there is also substantial evidence that perceptual competition arising from dichoptic visual stimulus presentation can occur at the level of stimulus representations, as opposed to strictly monocular representations (Logothetis, Leopold, & Sheinberg, 1996; Suzuki & Grabowecky, 2002; Ooi & He, 2003; Silver & Logothetis, 2004; de Weert et al., 2005)

For some dichoptic stimulus pairs, there are perceptual interpretations that are based on interocular grouping of information from each eye to generate a percept that is coherent and integrates information from the two eyes (Díaz-Caneja, 1928; Ngo et al., 2000; Kovács, Papathomas, Yang, & Fehér, 1996; Suzuki & Grabowecky, 2002; Lee & Blake, 2004; de Weert et al., 2005). Díaz-Caneja stimuli are particularly useful for studying the relationships among interocular competition and interocular grouping processes in conscious perception, as they give rise to two monocular perceptual interpretations (leftward- and rightward-pointing horseshoes), as well as two well-defined and coherent interocularly-grouped interpretations (concentric rings and horizontal lines) (Figure 3).

In the present study, we found that passive viewing of Díaz-Caneja stimuli produced substantial predominance of all four possible perceptual interpretations, with the concentric circles percept being particularly dominant (Figure 4). It could be that although Díaz-Caneja stimuli contain especially strong Gestalt grouping cues for both concentric circles and horizontal lines (continuity of contours, feature similarity, connectedness, proximity), there are additional Gestalt grouping cues that are stronger or exclusive to the concentric circles percept (common region, closure). Further research with systematic manipulation of grouping factors is needed to better understand the relevant factors that govern interocular competition versus interocular grouping in multistable perception.

### Effects of volitional control in binocular rivalry

Interocular grouping in binocular rivalry provides a window into the process of perceptual inference (Von Helmholtz, 1867), in which perception is actively constructed by the brain to generate an interpretation based on information conveyed by the sensory organs as well as a variety of top-down factors, including prior experiences and knowledge (Denison, Piazza, & Silver, 2011; Denison, Sheynin, & Silver, 2016; Lawler & Silver, 2023), Gestalt grouping (Alais & Blake, 1999; Sobel & Blake, 2002; Silver & Logothetis, 2004; de Weert et al., 2005), and attention (Chong, Tadin, & Blake, 2005; Hancock & Andrews, 2007; Paffen & Alais, 2011).

Volitional control is another example of a top-down contribution to the active construction of perceptual experience. For some types of multistable stimuli such as the Necker cube, subjects have been reported to have substantial volitional influence over which perceptual interpretation is dominant (George, 1936; Meng & Tong, 2004; van Ee et al., 2005). Moreover, participants can significantly influence the rate of alternation between the two monocular percepts in binocular rivalry (George, 1936; Lack, 1969; Meng & Tong, 2004; van Ee et al., 2005). However, there is much more limited volitional control over which of the competing percepts is dominant in conventional binocular rivalry (Meng & Tong, 2004; van Ee et al., 2005).

In the present study, we also found very limited volitional control of predominance for binocular rivalry between orthogonal gratings (Experiment 1; Figures 1 and 2). In contrast, participants could exert strong and reliable control over the contents of perception for each of the four possible coherent perceptual interpretations of Díaz-Caneja stimuli (Experiment 2; Figure 6). Interestingly, for these stimuli, volitional enhancement of the monocular horseshoe percepts was largely due to increases in the mean dominance duration of these percepts (prolonged maintenance of the percept once it has become dominant), while volitional enhancement of the interocularly-grouped percepts occurred mainly through an increase in the probability of selection (Figure 7).

### Possible confounding factors in measurement of volitional effects on perception

Because all of the behavioral responses in our study are based on participants’ subjective reports of their conscious perception, it is possible that task instructions to increase predominance of one of the perceptual interpretations could result in effects due to response bias. In our study, response bias could result from participants’ desire to effectively comply with the volitional instructions and could lead them to report higher predominance of the cued perceptual interpretation in a way that exceeded the actual effects of volitional control on their subjective perception.

To estimate possible effects of response bias, we conducted a control study (Experiment 3; Figure 8) in which one of the stimulus sets consisted of concentric circles presented to one eye and horizontal lines to the other eye. Like Díaz-Caneja stimuli, these stimuli afford four possible perceptual interpretations. However, unlike Díaz-Caneja stimuli, perceiving a leftward- or rightward-pointing horseshoe in our control experiment requires overcoming both Gestalt grouping cues and differences in monocular inputs. A similar approach was employed by Knapen et al. (2007) to characterize the effects of stimulus flicker on perceptual interpretations of Díaz-Caneja stimuli.

We reasoned that any response bias effects would be due to task instructions and should not be strongly affected by the ocular configuration of the rivalrous stimuli. Our results indicated minimal volitional effects when subjects were instructed to enhance perception of either leftward- or rightward-pointing horseshoes for the control stimulus set of concentric circles and horizontal lines. These findings suggest that response bias is unlikely to be a significant contributing factor to the pattern of results observed in Experiment 2.

Our study does not account for the possibility that subjects may have employed various eye movement strategies, including both conjugate and non-conjugate eye movements, to select or stabilize cued percepts. Although we did instruct participants to fixate their gaze on a central fixation point, we did not verify fixation with eye tracking measures. Future studies could incorporate eye tracking to ensure compliance with fixation instructions.

### Multiple levels of competition in multistable perception

The four perceptual interpretations afforded by Díaz-Caneja stimuli suggest both monocular (horseshoe percepts) and binocular (interocularly-grouped concentric circles and horseshoes) representations that compete with one another for perceptual dominance (Knapen et al., 2007). Besides Díaz-Caneja stimuli, a variety of other rivalrous stimuli can lead to unitary percepts based on interocular grouping of portions of the monocular inputs (Kovács et al., 1996; Suzuki & Grabowecky, 2002; Lee & Blake, 2004; de Weert et al., 2005).

Additionally, interocular switch (IOS) rivalry occurs when dichoptic stimuli are swapped between the two eyes at a rate of 3 Hz (Logothetis et al., 1996). Like Díaz-Caneja stimuli, IOS rivalry of orthogonal gratings can lead to percepts that are either monocular (3 Hz changes in perceived grating orientation that reflect one of the monocular inputs) or binocular (slow irregular changes in perceived orientation in which a given percept spans multiple interocular stimulus switches) (Denison & Silver, 2012; Logothetis et al., 1996; Silver & Logothetis, 2007). Although interocular grouping of Díaz-Caneja stimuli involves spatial integration of a unitary percept from components of the monocular inputs, IOS rivalry reflects temporal integration of a stable dominant percept over interocular stimulus switches.

Wilson (2003) constructed a two-stage hierarchical neural model with recurrent inhibition that accounts for both monocular and binocular perceptual interpretations of IOS rivalry of orthogonal gratings. An analogous model consisting of monocular competition early in visual processing and competition between binocular stimulus representations at higher levels of the visual processing hierarchy qualitatively accounts for the four perceptual interpretations of Díaz-Caneja stimuli and the fact that interocular grouping is needed to generate the percepts of concentric circles and horizontal lines.

### Distinct properties of perceptual maintenance and selection

We found significant effects of volitional enhancement on predominance of each of the four perceptual interpretations of Díaz-Caneja stimuli. These effects of volition occurred through two different mechanisms: enhancement of monocular horseshoe percepts was due to prolongation of the dominance durations of selected perceptual interpretations, whereas enhancement of interocularly-grouped concentric circles and horizontal lines resulted from increasing the probability of selection of those perceptual interpretations.

A number of studies have examined factors that differentially influence perceptual maintenance versus selection (reviewed in Bressler et al., 2013). For example, increasing the contrast of a currently dominant stimulus in binocular rivalry prolongs the mean duration of dominance for that stimulus (Chong et al., 2005; Mueller & Blake, 1989). This may indicate that perceptual maintenance in binocular rivalry reflects a relatively low-level process related to stimulus strength, possibly in early visual cortex. However, both of these studies used binocular rivalry of orthogonal gratings, and competition between these elemental stimuli may take place at earlier stages of the visual system than competition between more complex shapes or objects. Moreover, directing endogenous attention to a dominant grating also prolongs its mean dominance duration, indicating top-down modulation (Chong et al., 2005).

In general, perceptual grouping factors affect perceptual selection more than maintenance in binocular rivalry. This has been reported for collinearity (Alais & Blake, 1999; Alais et al., 2006), Gestalt cues for shape (de Weert et al., 2005), and color and luminance uniformity (Silver & Logothetis, 2004). These effects of grouping cues on perceptual selection in binocular rivalry are broadly consistent with our finding that volitional enhancement of interocularly-grouped percepts occurs through increased perceptual selection.

Although the neural bases of volitional effects on perceptual maintenance and selection are not currently known, the double dissociation of mechanisms that we report here is well-suited for future neuroscientific investigations in human subjects. Subjects can report their unitary percepts of Díaz-Caneja stimuli in real time during simultaneous recording of brain activity, thereby enabling robust measurement of brain/behavior correlations, and the four distinct perceptual interpretations are not confounded by changes in the visual stimuli. Additionally, the fact that volitional effects on perception of Díaz-Caneja stimuli can manifest through changes in either mean dominance duration or probability of perceptual selection allows for explicit comparison of patterns of brain activation associated with these two distinct components of conscious perception and its volitional control.

## Conclusions

In the present study, we replicated previous reports of subjects having very limited volitional control over the contents of conscious perception for conventional binocular rivalry between gratings with orthogonal orientations. In contrast, subjects exhibited substantial volitional control over perception of Díaz-Caneja stimuli, both for perceptual interpretations corresponding to one of the monocular stimuli and for those arising from interocular grouping across the two monocular stimuli. We observed a double dissociation of the mechanisms underlying this volitional control of perception of Díaz-Caneja stimuli. Enhancement of the monocular percepts occurred mainly through increased mean dominance duration, whereas enhancement of the interocularly-grouped percepts was primarily due to increased probability of perceptual selection. These results deepen our understanding of the processes by which the visual system creates coherent perceptual interpretations from ambiguous visual inputs.
